# The Editorial Position on ‘Recent Advances in Multifunctional Antimicrobial Peptides as Preclinical Therapeutic Studies and Clinical Future Applications’

**DOI:** 10.3390/pharmaceutics15102383

**Published:** 2023-09-26

**Authors:** Francesco Scavello, Mohamed Amiche, Jean-Eric Ghia

**Affiliations:** 1 IRCCS Humanitas Research Hospital, Rozzano, 20089 Milan, Italy; 2 Laboratoire de Biogenèse des Signaux Peptidiques (BioSiPe), Institut de Biologie Paris-Seine, Sorbonne Université-CNRS, 75252 Paris, France; 3 Department of Immunology, Rady Faculty of Health Sciences, University of Manitoba, Winnipeg, MB R3E 0T5, Canada; 4 Section of Gastroenterology, Department of Internal Medicine, Rady Faculty of Health Sciences, University of Manitoba, Winnipeg, MB R3E 0T5, Canada

Antibiotic resistance has recently been recognized as an alarming issue and one of the leading causes of death worldwide [1]. In the last few years, the frequent use of antibiotics in COVID-19 patients has exacerbated antimicrobial resistance [2]. Antimicrobial peptides (AMPs) are an alternative to antibiotic therapy, and they have a great potential to respond to multidrug-resistant pathogens [3]. They offer several advantages compared to classical antibiotics such as broad-spectrum activity and a lower tendency to develop resistance mechanisms and modulators of the host immune response [3]. Some of these molecules are named Multifunctional AMPs (MF-AMPs) due to their ability to influence several biological processes and their comprehensive antimicrobial function.

This Special Issue in *Pharmaceutics*, “Recent Advances in Multifunctional Antimicrobial Peptides as Preclinical Therapeutic Studies and Clinical Future Applications”, is in honor of Dr. Marie-Hélène Metz-Boutigue, acknowledging her influential scientific contributions to research on AMPs and highlighting the recent advances in MF-AMPs research. In biomedical research, there are few pioneers whose groundbreaking contributions leave an indelible mark on their field. Among these stands Dr. Metz-Boutigue, a visionary scientist whose work in the field of AMPs has revolutionized our understanding of some of them. Her collaborative efforts with esteemed researchers have ushered in a new era of potential treatments for infectious diseases. Dr. Metz-Boutigue’s interest in AMPs dates back to her early career when she recognized the immense therapeutic potential of these peptides. Her journey was marked by relentless dedication, determination, and a passion for unravelling the complexities of the mechanism of action of these peptides. Through her prolific research, Dr. Metz-Boutigue deepened our knowledge of AMPs and discovered novel classes of these peptides with potent antimicrobial activity. Her work paved the way for developing innovative therapeutic strategies against various infectious agents, including bacteria, fungi, and even viruses.

One of the significant aspects of Dr. Metz-Boutigue’s remarkable success has been her collaborative spirit, as she has fostered partnerships with brilliant researchers worldwide. Among her notable collaborators are Prof. Schneider, renowned for his expertise in intensive care medicine and infection, and together they discovered the role of several AMPs as biomarkers in critically ill patients [4]; Dr. Prévost, an expert in microbiology, who, together with Dr. Metz-Boutigue, identified new AMPs against resistant *S. aureus* strains [5]; and Dr. Goumon, an expert in neuroscience, with whom Dr. Metz-Boutigue discovered several AMPs derived from the neuroendocrine system [6]. Of note are her collaborations with Dr. Jollès and Dr. Strub, specialists in protein biochemistry and mass spectrometry, respectively, through which they characterized the biochemistry and processing of several proteins producing AMPs [7,8]. Concerning the findings demonstrating the multifunctional profile of AMPs, we mentioned the collaborations with Prof. Angel and Prof. Ghia, prominent gut physiologists, who, together with Dr. Metz-Boutigue, characterized the role of some AMPs in colonic motility [9]. Additionally of note are her collaborations with Prof. Corti and Prof. Helle, experts in biochemistry, who together have demonstrated the role of several AMPs derived from Chromogranins (Cgs) in different biological processes [10]. Concerning Cgs-derived peptides, Dr. Metz-Boutigue collaborated with Prof. Mahata, with whom she discovered the role of Catestatin as an antimicrobial agent [11], and with Dr. Marban-Doran and Prof. Rohr, with whom she identified new Chromogranin A (CgA)-derived AMPs and their role in the context of in nosocomial infections [12,13]. Also relevant are the studies conducted in collaboration with Prof. Aunis, a prominent neuroscientist, showing the role of neuropeptides in innate immunity [14], as well as her work in collaboration with Prof. Tota and Prof. Angelone, who are renowned for their expertise in cardiac physiology, demonstrating the role of AMPs in cardiac physiopathology [10]. In the last few years, Dr. Metz-Boutigue’s collaborations with Prof. Haikel, a specialist in oral biology, should be mentioned, which enabled the application study of AMPs in dental biomaterials and infections [15]. Finally, her fruitful collaborations with Dr. Lavalle and Prof. Schaaf, experts in biomaterials, enabled the development of functionalized biomaterials with antimicrobial and anti-inflammatory properties [16,17]. All these collaborations have synergized the researchers’ diverse knowledge and methodologies, resulting in transformative research outcomes. Beyond her academic achievements, the collaborative work of Dr. Metz-Boutigue has not only catalyzed the development of international partnerships in the battle against infectious diseases but has also helped the next generation of scientists in this field of research by undoubtedly reshaping the landscape of AMPs and suggesting the concept of MF-AMPs, notably for Cgs and their derived AMPs. As we look toward the future, we eagerly await the progress of the next generation in this field of research.

In this Special Issue, several manuscripts reported the ability of MF-AMPs as an alternative or adjuvant to classical antibiotics to overcome antibiotic resistance. In general, Hassan et al. recapitulate the positive aspects of these molecules, such as a broad spectrum of antimicrobial activities, high pathogen specificity, and low toxicity for the host. In addition, they summarized the sources, structure, molecular mechanisms of action, and therapeutic potential of AMP, evaluating the advantages over classical antibiotics such as a low propensity for the development of resistance, endogenous origins, and anti-biofilm activity. However, they reported several limitations, such as poor absorption and distribution, a short half-life, low permeability, and some toxic effects [18].

Regarding the resistance problem, Kuhn and Di found that several pathogens were resistant to last-report antibiotic peptides classically used for multidrug-resistant bacterial treatment, such as Colistin. They demonstrated the importance of mutation timing in the progression of the *Klebsiella pneumoniae* Colistin-resistant strain, identifying a time-specific mutation of Colistin-resistant genes during this pathogen evolution. In particular, the authors observed the onset of an early resistance with mutations relevant to capsule production, cell membrane integrity, and energy metabolism in response to Colistin treatment, followed by mutations influencing LPS structure, strongly impacting Colistin-resistant progression [19].

Also, different studies are presented that dealt with improving antimicrobial activities and reducing their adverse effects using structure–function relationship studies, engineered variations, chemical modifications, and nanoparticle incorporation [20,21,22]. To identify the structural requirements of AMP activity directed against Gram-negative bacteria, Madi-Moussa and collaborators heterologously produced a series of variants of Lacticaseicin 30 including the N-terminal (amino acids 1 to 39) and central and C-terminal (amino acids 40 to 111) domains in *E. coli* and assayed their antibacterial activities. In addition, ten mutations were introduced to obtain several engineered variants. In comparison to the original AMP, they observed no differences for the N-terminal peptide and variants E32G, T33P, and D57G preserving the antibacterial effects, but demonstrated a reduced antimicrobial activity against Gram-negative bacteria for the C-terminal peptide and the E6G, T7P, T52P, A74P, Y78S, Y93S, and A97P variants [20]. Finally, the authors synthesized an N terminal domain without the first 20 amino acids in the first helix, showing that this truncated peptide did not have antimicrobial activity [20]. Another approach used by Etayash et al. involved chemically modifying AMP IDR1018 by covalently linking it with a short-chain PEG (PEG6) and a glucose moiety (N-acetyl glucosamine: GlcNAc) and assessing the impact of this chemical modification on the peptide’s antimicrobial and biological properties. The pegylation and glycosylation significantly reduced the aggregation, hemolysis, and cytotoxicity effect of IDR1018 [21]. Of interest, PEG6-IDR1018 maintained the immunomodulatory activity of IDR1018 while the Glc-IDR1018 significantly increased the anti-inflammatory effects of this peptide (production of MCP-1 and IL1-RA *vs.* lipopolysaccharide-induced IL-1β) [21]. On the other hand, these chemical modifications partially reduced the antimicrobial and anti-biofilm activity [21]. To deliver the antimicrobial Esc peptides specifically into the lung, Cappiello and coworkers developed engineered poly(lactide-co-glycolide) (PLGA) nanoparticles (NPs). They demonstrated that the peptides alone and the nanoparticle version did not affect lung epithelium integrity. No changes in pulmonary global gene expression profile were observed after the treatments [22]. The Esc diastereomer showed the highest antimicrobial activity without lung inflammation; however, the nanoparticulate system increased the pulmonary safety and delivery of Esc peptides, prolonging the biostability of these peptides in the mouse bronchoalveolar lavage [22]. 

In this Special Issue, we included several studies that also reported the possible applications of MF-AMPs in different human and clinical contexts. Some of these applications concern their antimicrobial nature, while others extend to new research fields. Jati and coworkers suggested that the pleiotropic CgA-derived peptide Catestatin is a perfect example of an MF-AMP, reporting its potential therapeutics in antimicrobial and immunological applications. They summarized this peptide’s antibacterial, antifungal, and anti-yeast activity and its N-terminal domain of 15 amino acids called Cateslytin. The author reported the chemically modified isoform for this smaller peptide, with the total D-amino acids changing, and its increased antimicrobial effects against various bacterial and fungal strains [23]. Finally, they dealt with the human variants of Catestatin (Gly364Ser and Pro370Leu) and the evolutionary conservation of this peptide in mammals, pointing out the potential role of this peptide as a therapy for antibiotic-resistant superbugs and its role in innate immunity and the regulation of gut microbiota [23]. Also, Rizzetto and collaborators recapitulated the experience of their research group over 20 years in a narrative review describing the antibacterial and antifungal properties of several AMPs such as BMAP-28, Citropin 1.1, Temporin A, Distinctin, Magainin II, LL-37, Tritrpticin, Colistin, Distinctin, Magainin II, Cecropin A, and many others [24]. This review strongly supports the benefits of these molecules, suggesting AMPs as a valuable alternative for treating infectious diseases related to multiresistant microorganisms and overcoming the clinical issue of resistance against the commonly used antibiotics or antifungals. 

One of the most relevant applications for AMPs in biomedical devices is in the field of biomaterials due to the anti-biofilm properties of these molecules [24]. As reported in many in vitro and in vivo studies, these molecules are elective factors for oral infection and biomaterials to treat early carious lesions, promote cell adhesion, and enhance the adhesion strength of dental implants while preventing infection at the surgery site [25]. However, the small amount of data regarding animal models or clinical trials is a limitation for the future use of this biomaterial containing AMPs [25]. In this Special Issue, Cunha and coworkers developed a clinical trial to evaluate in vivo the antimicrobial properties and dental efficacy of a nisin-biogel in dogs [26]. This study reported that the nisin-biogel group was associated with a significantly reduced periodontal pocket depth and dental plaque index after 90 days from the treatment compared to control animals. Also, a non-significant reduction in the gingivitis index was observed, while no influence on total bacterial counts and adverse effects was detected. Finally, the author suggested the nisin–biogel system as a valuable biomaterial for future human dentistry study. In addition, the crucial role of AMPs in the reduction of endodontic inflammation and infection was demonstrated by Morio et al. They, evaluating different datasets of ultraviolet (UV)-induced molecules from other tissues, such as endodontic tissue, identified 32 UV-induced molecules containing nine antimicrobial peptides (Cathelicidin and several Defensins), ten cytokines, six growth factors, three enzymes, two transmembrane receptors, and two transcription regulators. Many of these molecules are related to the wound-healing signaling pathway and communication between immune cells and antimicrobial response. They concluded that, also via the UV irradiation technique, AMPs production requires part of the activate innate immune response with a final aim to reduce infection but also assist tissue healing [27].

Another important application for MF-AMPs in human health is their roles as the biomarkers of several diseases both related and unrelated to infections [28,29,30,31]. In this Special Issue, Scnheider and collaborators summarized the biomarker profile of CgA and its derived antimicrobial peptides in the context of critically ill patients [4]. They reported the role of CgA as a biomarker of outcome in non-selected critically ill patients. Then, they focused on Vasostatin-I, an N-terminal fragment of CgA, and its ability as an early prognostic biomarker for these patients associated with age and lactate. Also, for trauma patients, CgA levels can predict the onset of nosocomial infections, and their CgA-derived peptides can act as inhibitors of inflammation and antimicrobial peptides. Finally, they also demonstrated that a 4% concentration of non-oxidized albumin infusion reverses the multimerization of these peptides, restoring their anti-inflammatory and antimicrobial properties [4]. They concluded that CgA and its peptides could be considered relevant biomarkers in critically ill patients. In addition, Scavello and coworkers reported the role of the multifunctional agents of CgA-derived AMPs, describing these as endogenous modulators of inflammation [32]. Concerning MF-AMPs and cancer, Dermaseptins, a class of α-helical-shaped polycationic peptides isolated from the skin secretions of several leaf frogs, have been identified as examples of exogenous molecules characterized by their anticancer activity [32].

Finally, Neuropilin-1 and Integrins have been recently described by Corti et al. as receptors with a high affinity for CgA-derived peptides [33]. In particular, the multifunctional properties of this protein and its fragments reported in angiogenesis, wound healing, and tumor growth are mediated by a specific interaction and binding of Neuropilin-1 and Integrin avb6 [33]. Moreover, Siegel and collaborators described an immunogenicity test for dendritic and CD4-positive T cells where different peptides and proteins have been used to stimulate both dendritic and CD4-positive cells [34], suggesting that MF-AMPs may be used in the future in this context to assess immunogenicity as well.

In summary, this research topic has brought to light new findings regarding MF-AMPs and their possible applications, as well as future studies, for human health. 
